# Supplementary material for: Comparative Genomic Analyses of the Genus Photobacterium Illuminate Biosynthetic Gene Clusters Associated with Antagonism
Source: Int J Mol Sci. 2022 Aug 26;23(17):9712. doi: 10.3390/ijms23179712 (PMC9456166; doi:10.3390/ijms23179712)
Supplement: Supplementary file 1 [file ijms-23-09712-s001.zip › ijms-1860717-supplementary.pdf]

Supplementary Materials

Comparative Genomic Analyses of the Genus *Photobacterium* Illuminate Biosynthetic Gene Clusters Associated with Antagonism

Nyok-Sean Lau[1], Wooi Liang Heng[1], Noorizan Miswan[1], Nor Azura Azami[1] and Go Furusawa[1,*]

[1]Centre for Chemical Biology, Universiti Sains Malaysia, Penang, Malaysia

*Correspondence: furusawa@usm.my

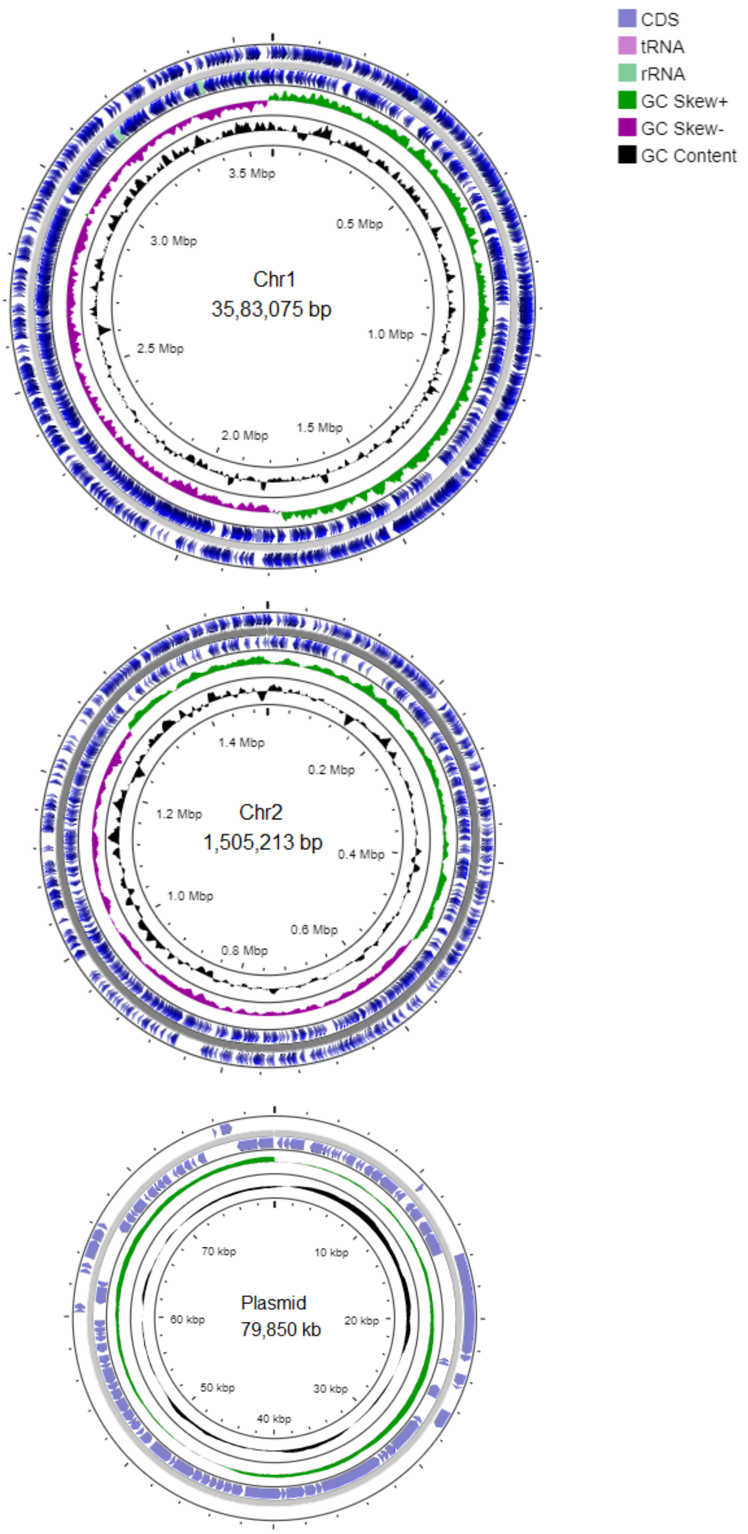

Figure S1. Circular representation of *Photobacterium* sp. CCB-ST2H9 genome. The circles display (from the outside): CDS, tRNA, rRNA, GC skew and GC content. The genome map was generated using Proksee (proksee.ca).

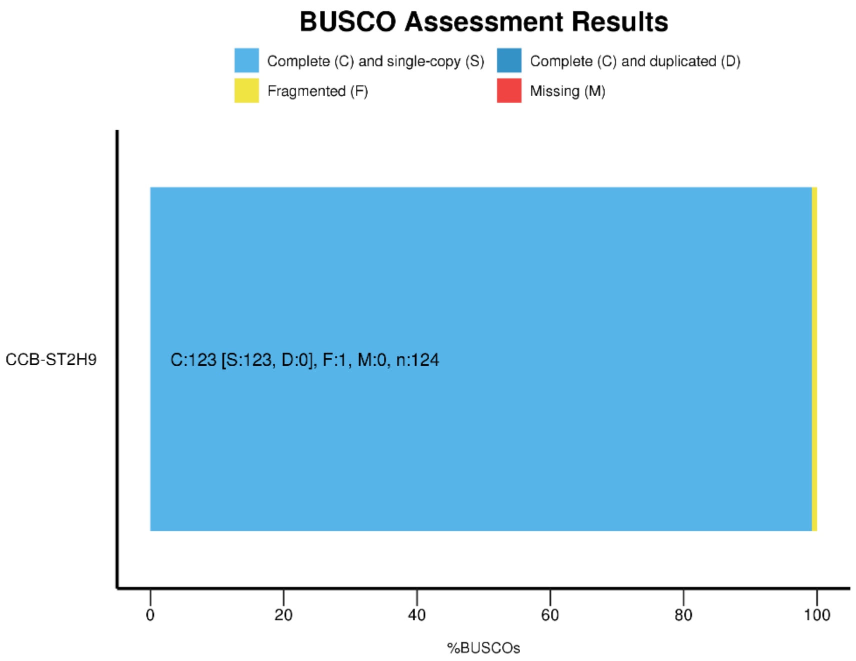

Figure S2. BUSCO completeness assessments of *Photobacterium* sp. CCB-ST2H9 genome. The blue, light blue, dark blue, yellow, and red bars represent the proportion of complete (C), complete single-copy (S), complete duplicated (D), fragmented (F), and missing (M) BUSCO genes, respectively.

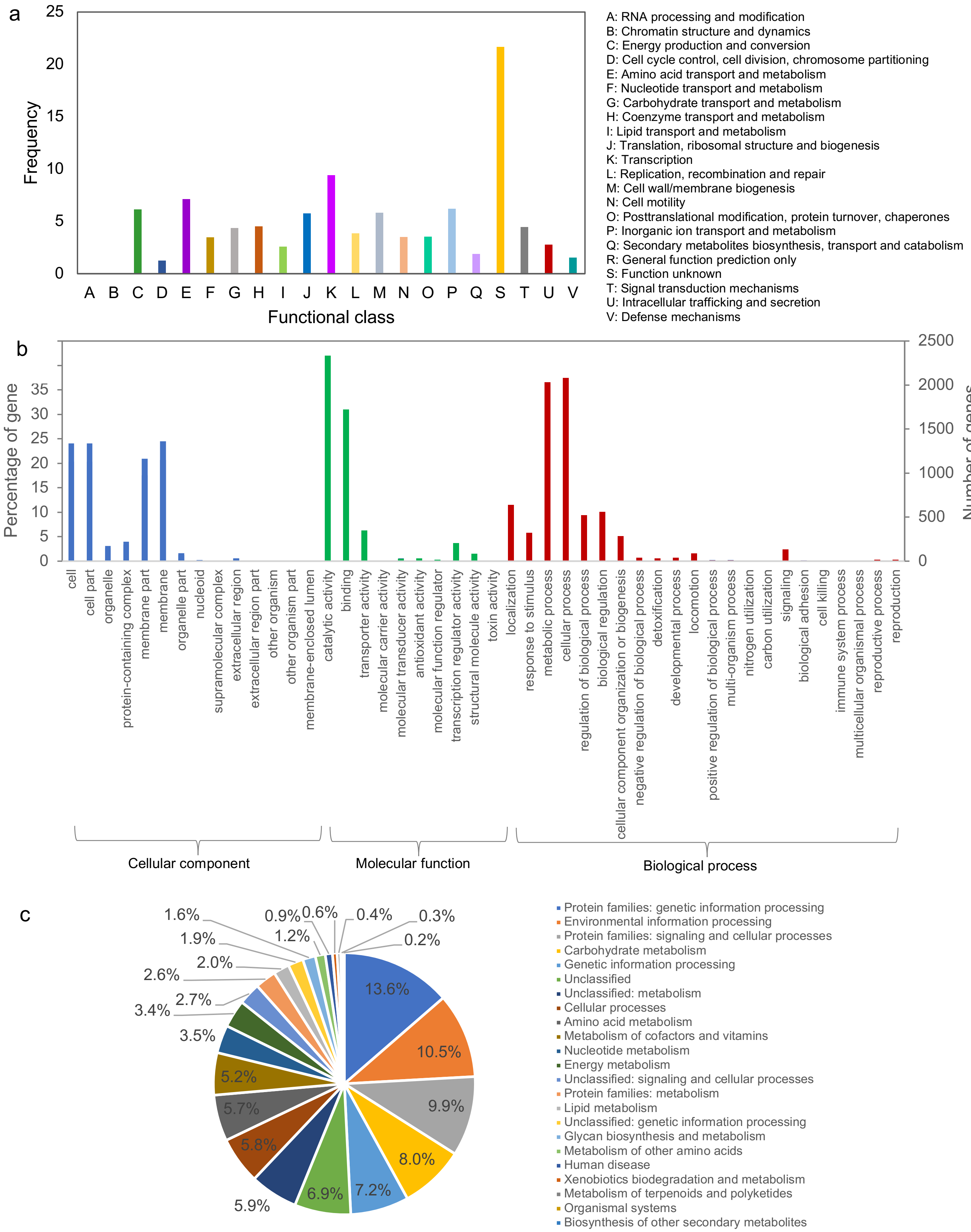

Figure S3. Functional classifications of CCB-ST2H9 genes to (A) COG, (B) GO and (C) KEGG databases, respectively.

Table S1. Genomes of *Photobacterium* used in the analyses. Genomes of type strains from species in the genus *Photobacterium* were downloaded from NCBI GenBank. If genome of the type strain was not available, we then selected assembly with the highest completion level.

Strain[a]	Genome size (Mb)	GC (%)	Genes	GenBank accession no.	Origin	Isolation site characteristic	Reference
P. alginatilyticum P03D4	6.53	47.9	5,828	NZ_RSEJ01000001	East China Sea	Bottom seawater	Wang et al. [1]
P. andalusiense CECT 9192	4.46	39.4	4,105	NZ_FYAJ00000000	Spain	Diseased redbanded seabream	Labella et al. [2]
P. angustum ATCC 25915	4.97	39.6	4,498	PYOM01000001	International waters	Seawater at depth of 750 m	Reichelt et al. [3]
P. aphoticum KCTC 23057	5.25	49.7	4,666	NZ_BMYC01000001	Spain	Surface seawater	Lucena et al. [4]
P. aquae CGMCC1.12159	5.08	49.1	4,652	NZ_LDOT01000001	China	Grouper culture tank	Liu et al. [5]
P. aquimaris LC2-065	4.53	39.5	4,054	NZ_MSCC01000001	Japan	Seawater	Yoshizawa et al. [6]
P. arenosum CAU 1568	4.78	50.1	4,370	NZ_JACYTP010000001	Korea	Marine sediment sand	Weerawongwiwat et al. [7]
P. carnosum TMW2.2021	4.32	38.6	3,957	NZ_NPIB01000001	Germany	Spoiled poultry meat	Hilgarth et al. [8]
P. chitinilyticum BE1247	5.92	46.5	5,326	NZ_RJLM01000001	East China Sea	Bottom seawater	Wang et al. [9]
P. damselae subsp. *damselae* KC-Na-1	4.54	40.9	4,257	NZ_CP021151	South Sea	Finless porpoise	Lee et al. [10]
P. frigidiphilum JCM 12947	6.45	41.6	6,023	NZ_PYMJ01000001	Pacific Ocean	Dee-sea sediments	Seo et al. [11]
P. gaetbulicola Gung47	5.91	49.7	5,277	NZ_CP005973	Korea	Tidal flat sediment	Kim et al. [12]
P. galatheae S2753	4.53	49.5	4,121	NZ_JMIB01000001	Solomon sea	Mussel	Machado et al. [13]
P. ganghwense C2.2	5.74	50.3	5,230	NZ_CP071325	Black sea	Wet sand	Lascu et al. [14]
P. halotolerans DSM 18316	4.69	50.9	4,283	NZ_KE38431	Spain	Saline lake	Rivas et al. [15]
P. iliopiscarium ATCC 51760	4.31	39.0	3,875	NZ_JZSQ01000001	Norway	Herring pyloric ceca	Urakawa et al. [16]
P. indicum ATCC 19614	5.91	41.5	5,322	NZ_PYOC01000001	India Ocean	Marine mud	Xie and Yokota [17]
P. jeanii R-40508	5.05	45.1	4,508	NZ_LVHF01000001	Brazil	Mucus of *Palythoa caribaeorum*	Chimetto et al. [18]

P. kishitanii pjapo1.1	4.73	39.1	4,273	NZ_PYNK01000001	Japan	Light organ of *Physiculus japonicus*	Ast et al. [19]
P. leiognathi ATCC 25221	4.63	40.9	4,228	NZ_JZSK01000001	Malaysia	Light organ of *Leiognathus equula*	GenBank
P. leiognathi subsp. *Mandapamensis* svers.1.1.	4.60	41.1	4,071	NZ_PYLZ01000001	N/A	Light organ of *Siphamia versicolor*	Urbanczyk et al. [20]
P. lipolyticum DSM 16190	4.94	45.9	4,421	NZ_PYMC01000001	Korea	Intertidal sediment	Yoon et al. [21]
P. lucens CAIM 1937	4.51	41.4	4,073	NZ_SSMG01000001	Mexico	Hepatopancreas of *Penaeus vannamei*	Enciso-Ibarra et al. [22]
P. lutimaris JCM 13586	5.97	47.6	5,351	NZ_PYMH01000001	Korea	Tidal flat sediment	Jung et al. [23]
P. malacitanum CECT 9190	4.38	39.8	4,006	NZ_FYAK01000055	Spain	Diseased redbanded seabream	Labella et al. [2]
P. marimum AK15	5.54	46.2	4,879	NZ_AMZO01000001	India	Marine sediment	Srinivas et al. [24]
P. phosphoreum JCM 21184	4.55	39.8	3,973	NZ_MSCQ01000001	N/A	Marine fish skin	GenBank
P. piscicola NCB100098	4.53	39.2	4,207	NZ_FUZI01000061	Netherlands	Whiting skin	Figge et al. [25]
P. profundum SS9	6.40	41.7	5,860	NC_006370	Sulu Sea	Amphipod at depth of 2551 m	Delong et al. [26]
P. proteolyticum 13-12T	6.48	47.9	5,768	NZ_MJIL01000001	China	Ocean sediment	Li et al. [27]
P. rosenbergii DSM 19138	6.35	47.6	5,853	NZ_PYMB01000001	Australia	Bleached coral	Thompson et al. [28]
P. salinisoli LAM9072	4.74	50.2	4,393	NZ_QZMS00000000	China	Saline soil	Li et al. [29]
P. sanctipauli DSM 100436	5.84	47.9	5,285	NZ_PYMA01000001	Brazil	Bleached coral	Moreira et al. [30]
P. sanguinicancri CAIM 1827	5.48	43.7	4,974	NZ_NOIF01000001	Spain	Hemolymph of spider crab	Gomez-Gil et al. [31]
P. swingsii DSM 24669	5.54	43.7	4,930	NZ_PYLZ01000001	Spain	Hemolymph of spider crab	Gomez-Gil et al. [32]
P. *toruni* CECT 9198	4.42	38.6	4,183	NZ_FUWP01000118	Spain	Spleen of diseased redbanded seabream	Labella et al. [33]

Table S2. Genomic islands (GIs) detected in the CCB-ST2H9 genome by IslandViewer4 [34].

Location	Start	End	Length (bp)	Detection Method
Contig 1	780,386	813,447	33,061	IslandPath-DIMOB
Contig 1	965,651	982,966	17,315	SIGI-HMM
Contig 1	1,513,192	1,526,788	13,596	SIGI-HMM
Contig 1	2,462,369	2,490,079	27,710	IslandPath-DIMOB
Contig 1	3,479,455	3,504,073	24,618	IslandPath-DIMOB
Contig 2	3,719,497	3,730,896	11,399	SIGI-HMM
Contig 2	4,561,321	4,597,109	35,788	IslandPath-DIMOB
Contig 2	4,623,293	4,633,504	10,211	SIGI-HMM
Contig 2	4,822,763	4,835,102	12,339	SIGI-HMM
Contig 3	5,093,778	5,105,547	11,769	SIGI-HMM
Contig 3	5,113,360	5,135,615	22,255	SIGI-HMM
Contig 3	5,140,212	5,151,187	10,975	SIGI-HMM
Contig 3	5,153,507	5,164,127	10,620	SIGI-HMM

Table S3. Genomic islands detected in the CCB-ST2H9 genome.

Island start	Island end	Length	Locus	Gene start	Gene end	Strand	Gene product name
780386	813447	33061	L4174_02616	780386	781279	-1	hypothetical protein
780386	813447	33061	L4174_02615	781686	782342	1	hypothetical protein
780386	813447	33061	L4174_02614	782390	782881	-1	hypothetical protein
780386	813447	33061	L4174_02613	782892	783356	-1	hypothetical protein
780386	813447	33061	L4174_02612	783508	783708	1	hypothetical protein
780386	813447	33061	L4174_02611	783731	784174	-1	hypothetical protein
780386	813447	33061	L4174_02610	784221	784649	-1	hypothetical protein
780386	813447	33061	L4174_02609	784820	784963	1	hypothetical protein
780386	813447	33061	L4174_02608	785193	787451	1	hypothetical protein
780386	813447	33061	L4174_02607	787613	789268	-1	hypothetical protein
780386	813447	33061	L4174_02606	789463	790992	1	hypothetical protein
780386	813447	33061	L4174_02605	790982	791395	1	hypothetical protein
780386	813447	33061	L4174_02604	791488	793611	1	hypothetical protein
780386	813447	33061	L4174_02603	793611	795098	1	hypothetical protein
780386	813447	33061	L4174_02602	795098	796435	1	hypothetical protein
780386	813447	33061	L4174_02601	796435	800754	1	hypothetical protein
780386	813447	33061	L4174_02600	800754	801581	1	hypothetical protein
780386	813447	33061	L4174_02599	801578	802711	1	hypothetical protein
780386	813447	33061	L4174_02598	802714	804399	1	hypothetical protein
780386	813447	33061	L4174_02597	804392	804967	1	hypothetical protein
780386	813447	33061	L4174_02596	804967	806502	1	Serine/threonine-protein kinase PknD
780386	813447	33061	L4174_02595	806907	809513	1	hypothetical protein
780386	813447	33061	L4174_02594	809513	810811	1	hypothetical protein
780386	813447	33061	L4174_02593	810907	811104	1	hypothetical protein
780386	813447	33061	L4174_02592	811148	812386	-1	Prophage integrase IntA
780386	813447	33061	L4174_02590	812965	813447	-1	SsrA-binding protein
965651	982966	17315	L4174_02453	965651	966790	1	hypothetical protein
965651	982966	17315	L4174_02452	966794	967234	1	putative low molecular weight protein-tyrosine-phosphatase AmsI
965651	982966	17315	L4174_02451	967333	969492	1	Tyrosine-protein kinase wzc
965651	982966	17315	L4174_02450	969540	970607	1	dTDP-glucose 4,6-dehydratase 2
965651	982966	17315	L4174_02449	970607	971482	1	Glucose-1-phosphate thymidylyltransferase 2
965651	982966	17315	L4174_02448	971488	972939	1	hypothetical protein
965651	982966	17315	L4174_02447	972936	974039	1	dTDP-4-amino-4,6-dideoxy-D-glucose transaminase
965651	982966	17315	L4174_02446	974027	974707	1	2,3,4,5-tetrahydropyridine-2,6-dicarboxylate N-acetyltransferase
965651	982966	17315	L4174_02445	974704	975708	1	Carbamoyl-phosphate synthase large chain
965651	982966	17315	L4174_02444	975715	976746	1	hypothetical protein
965651	982966	17315	L4174_02443	976749	977924	1	hypothetical protein
965651	982966	17315	L4174_02442	977921	978499	1	Galactoside O-acetyltransferase
965651	982966	17315	L4174_02441	978499	979647	1	hypothetical protein
965651	982966	17315	L4174_02440	979658	980665	1	UDP-N-acetylglucosamine 4-epimerase
965651	982966	17315	L4174_02439	980673	981785	1	N, N'-diacetylbacillosaminyl-diphospho-undecaprenol alpha-1,3-N-acetylgalactosaminyltransferase
965651	982966	17315	L4174_02438	981846	982493	1	UDP-glucose 6-dehydrogenase

965651	982966	17315	L4174_02437	982640	982966	1	UDP-glucose 6-dehydrogenase
1513192	1526788	13596	L4174_01957	1513192	1513542	1	hypothetical protein
1513192	1526788	13596	L4174_01956	1513673	1514104	1	hypothetical protein
1513192	1526788	13596	L4174_01955	1514192	1514698	1	Cytidylate kinase
1513192	1526788	13596	L4174_01954	1514834	1515223	1	hypothetical protein
1513192	1526788	13596	L4174_01953	1515368	1515763	1	hypothetical protein
1513192	1526788	13596	L4174_01952	1515898	1516125	1	hypothetical protein
1513192	1526788	13596	L4174_01951	1516226	1516690	1	putative N-acetyltransferase YafP
1513192	1526788	13596	L4174_01950	1516826	1517287	1	hypothetical protein
1513192	1526788	13596	L4174_01949	1517465	1517827	1	hypothetical protein
1513192	1526788	13596	L4174_01948	1517974	1518801	1	Hydroxyacylglutathione hydrolase
1513192	1526788	13596	L4174_01947	1519296	1519409	1	hypothetical protein
1513192	1526788	13596	L4174_01946	1519709	1520269	1	hypothetical protein
1513192	1526788	13596	L4174_01945	1520244	1520429	1	hypothetical protein
1513192	1526788	13596	L4174_01944	1520487	1520897	1	hypothetical protein
1513192	1526788	13596	L4174_01943	1521035	1521406	1	hypothetical protein
1513192	1526788	13596	L4174_01942	1521586	1522029	1	hypothetical protein
1513192	1526788	13596	L4174_01941	1522115	1522483	1	hypothetical protein
1513192	1526788	13596	L4174_01940	1522588	1522875	1	hypothetical protein
1513192	1526788	13596	L4174_01939	1522928	1523422	1	Acetyltransferase
1513192	1526788	13596	L4174_01938	1523566	1523985	1	hypothetical protein
1513192	1526788	13596	L4174_01937	1524137	1524787	1	hypothetical protein
1513192	1526788	13596	L4174_01936	1524920	1525624	1	L-aspartate/glutamate-specific racemase
1513192	1526788	13596	L4174_01935	1525772	1526251	1	hypothetical protein
1513192	1526788	13596	L4174_01934	1526375	1526788	1	hypothetical protein
2462369	2490079	27710	L4174_01101	2462369	2463448	-1	hypothetical protein
2462369	2490079	27710	L4174_01100	2463587	2463706	-1	hypothetical protein
2462369	2490079	27710	L4174_01099	2463833	2464249	-1	hypothetical protein
2462369	2490079	27710	L4174_01098	2464249	2464644	-1	hypothetical protein
2462369	2490079	27710	L4174_01097	2464631	2464978	-1	hypothetical protein
2462369	2490079	27710	L4174_01096	2464975	2465472	-1	hypothetical protein
2462369	2490079	27710	L4174_01095	2465465	2466286	-1	hypothetical protein
2462369	2490079	27710	L4174_01094	2466290	2466748	-1	hypothetical protein
2462369	2490079	27710	L4174_01093	2466750	2467022	-1	hypothetical protein
2462369	2490079	27710	L4174_01092	2467019	2467201	-1	hypothetical protein
2462369	2490079	27710	L4174_01091	2467204	2467470	-1	hypothetical protein
2462369	2490079	27710	L4174_01090	2467480	2468379	-1	hypothetical protein
2462369	2490079	27710	L4174_01089	2468382	2468507	-1	hypothetical protein
2462369	2490079	27710	L4174_01088	2468638	2469420	-1	hypothetical protein
2462369	2490079	27710	L4174_01087	2469383	2469895	-1	hypothetical protein
2462369	2490079	27710	L4174_01086	2469861	2470262	-1	hypothetical protein
2462369	2490079	27710	L4174_01085	2470348	2470656	-1	hypothetical protein
2462369	2490079	27710	L4174_01084	2470656	2472437	-1	hypothetical protein
2462369	2490079	27710	L4174_01083	2472437	2472625	-1	hypothetical protein
2462369	2490079	27710	L4174_01082	2472637	2472873	-1	hypothetical protein
2462369	2490079	27710	L4174_01081	2472986	2473753	1	hypothetical protein
2462369	2490079	27710	L4174_01080	2473750	2474745	1	Tyrosine recombinase XerC
2462369	2490079	27710	L4174_01079	2474895	2475311	-1	A-adding tRNA nucleotidyltransferase
2462369	2490079	27710	L4174_01078	2475462	2476781	-1	hypothetical protein
2462369	2490079	27710	L4174_01077	2476959	2477951	1	D-lactate dehydrogenase
2462369	2490079	27710	L4174_01076	2478141	2478689	1	hypothetical protein

2462369	2490079	27710	L4174_01075	2478826	2479698	-1	Succinate--CoA ligase [ADP-forming] subunit alpha
2462369	2490079	27710	L4174_01074	2479701	2480867	-1	Succinate--CoA ligase [ADP-forming] subunit beta
2462369	2490079	27710	L4174_01073	2480996	2482210	-1	Dihydrolipoyllysine-residue succinyltransferase component of 2-oxoglutarate dehydrogenase complex
2462369	2490079	27710	L4174_01072	2482243	2485056	-1	2-oxoglutarate dehydrogenase E1 component
2462369	2490079	27710	L4174_01071	2485156	2485869	-1	Succinate dehydrogenase iron-sulfur subunit
2462369	2490079	27710	L4174_01070	2485884	2487650	-1	Succinate dehydrogenase flavoprotein subunit
2462369	2490079	27710	L4174_01069	2487651	2487998	-1	Succinate dehydrogenase hydrophobic membrane anchor subunit
2462369	2490079	27710	L4174_01068	2487992	2488372	-1	Succinate dehydrogenase cytochrome b556 subunit
2462369	2490079	27710	L4174_01067	2488790	2490079	1	Citrate synthase
3479455	3504073	24618	L4174_00096	3479455	3479802	-1	hypothetical protein
3479455	3504073	24618	L4174_00095	3480481	3480816	1	hypothetical protein
3479455	3504073	24618	L4174_00094	3481384	3481623	1	hypothetical protein
3479455	3504073	24618	L4174_00093	3481865	3482332	1	hypothetical protein
3479455	3504073	24618	L4174_00092	3482410	3483609	-1	hypothetical protein
3479455	3504073	24618	L4174_00091	3483602	3484393	-1	hypothetical protein
3479455	3504073	24618	L4174_00090	3484396	3487674	-1	hypothetical protein
3479455	3504073	24618	L4174_00089	3487674	3488318	-1	hypothetical protein
3479455	3504073	24618	L4174_00088	3488302	3489639	-1	DNA replication and repair protein RecF
3479455	3504073	24618	L4174_00087	3489705	3490976	-1	hypothetical protein
3479455	3504073	24618	L4174_00086	3490966	3492483	-1	hypothetical protein
3479455	3504073	24618	L4174_00085	3492487	3492690	-1	hypothetical protein
3479455	3504073	24618	L4174_00084	3492747	3493004	1	hypothetical protein
3479455	3504073	24618	L4174_00083	3493215	3494204	1	hypothetical protein
3479455	3504073	24618	L4174_00082	3494208	3495128	1	hypothetical protein
3479455	3504073	24618	L4174_00081	3495140	3496552	-1	hypothetical protein
3479455	3504073	24618	L4174_00080	3496559	3497596	-1	hypothetical protein
3479455	3504073	24618	L4174_00079	3497756	3498010	-1	hypothetical protein
3479455	3504073	24618	L4174_00078	3498000	3500180	-1	Transposon Tn7 transposition protein TnsB
3479455	3504073	24618	L4174_00077	3500149	3500976	-1	hypothetical protein
3479455	3504073	24618	L4174_00076	3501101	3502288	-1	hypothetical protein
3479455	3504073	24618	L4174_00075	3502431	3502769	-1	Universal stress protein B
3479455	3504073	24618	L4174_00074	3502881	3503408	-1	putative bacterial non-heme ferritin
3479455	3504073	24618	L4174_00073	3503651	3504073	1	Universal stress protein A
3719497	3730896	11399	L4174_03457	3719497	3720858	1	hypothetical protein
3719497	3730896	11399	L4174_03458	3720872	3722092	1	hypothetical protein
3719497	3730896	11399	L4174_03459	3722085	3726806	1	Peptidoglycan-associated lipoprotein
3719497	3730896	11399	L4174_03460	3728370	3729578	1	hypothetical protein
3719497	3730896	11399	L4174_03461	3729709	3730896	1	hypothetical protein
4561321	4597109	35788	L4174_04263	4561321	4562085	-1	hypothetical protein
4561321	4597109	35788	L4174_04264	4562076	4562762	-1	hypothetical protein
4561321	4597109	35788	L4174_04265	4562759	4563298	-1	hypothetical protein
4561321	4597109	35788	L4174_04266	4563301	4566459	-1	hypothetical protein

4561321	4597109	35788	L4174_04267	4566467	4567063	-1	hypothetical protein
4561321	4597109	35788	L4174_04268	4567056	4568243	-1	hypothetical protein
4561321	4597109	35788	L4174_04269	4568236	4568559	-1	hypothetical protein
4561321	4597109	35788	L4174_04270	4568556	4570583	-1	hypothetical protein
4561321	4597109	35788	L4174_04271	4570772	4571035	-1	hypothetical protein
4561321	4597109	35788	L4174_04272	4571032	4571307	-1	hypothetical protein
4561321	4597109	35788	L4174_04273	4571308	4571829	-1	hypothetical protein
4561321	4597109	35788	L4174_04274	4571834	4572256	-1	hypothetical protein
4561321	4597109	35788	L4174_04275	4572253	4572474	-1	hypothetical protein
4561321	4597109	35788	L4174_04276	4572484	4572933	-1	hypothetical protein
4561321	4597109	35788	L4174_04277	4572933	4574054	-1	hypothetical protein
4561321	4597109	35788	L4174_04278	4574077	4574745	-1	hypothetical protein
4561321	4597109	35788	L4174_04279	4574723	4575205	-1	hypothetical protein
4561321	4597109	35788	L4174_04280	4575205	4575612	-1	hypothetical protein
4561321	4597109	35788	L4174_04281	4575721	4576431	-1	hypothetical protein
4561321	4597109	35788	L4174_04282	4576441	4577448	-1	hypothetical protein
4561321	4597109	35788	L4174_04283	4577449	4578351	-1	hypothetical protein
4561321	4597109	35788	L4174_04284	4578531	4580321	1	hypothetical protein
4561321	4597109	35788	L4174_04285	4580318	4581361	1	hypothetical protein
4561321	4597109	35788	L4174_04286	4581864	4582172	1	Endoribonuclease HigB
4561321	4597109	35788	L4174_04287	4582215	4582511	1	putative HTH-type transcriptional regulator YbaQ
4561321	4597109	35788	L4174_04288	4582643	4583128	-1	hypothetical protein
4561321	4597109	35788	L4174_04289	4583079	4583417	-1	hypothetical protein
4561321	4597109	35788	L4174_04290	4583624	4584034	1	hypothetical protein
4561321	4597109	35788	L4174_04291	4584066	4584308	1	hypothetical protein
4561321	4597109	35788	L4174_04292	4584618	4584863	1	hypothetical protein
4561321	4597109	35788	L4174_04293	4584907	4585023	1	hypothetical protein
4561321	4597109	35788	L4174_04294	4585164	4585409	1	hypothetical protein
4561321	4597109	35788	L4174_04295	4585563	4586003	1	hypothetical protein
4561321	4597109	35788	L4174_04296	4586000	4586266	-1	hypothetical protein
4561321	4597109	35788	L4174_04297	4586427	4586693	-1	hypothetical protein
4561321	4597109	35788	L4174_04298	4586690	4586857	-1	hypothetical protein
4561321	4597109	35788	L4174_04299	4586873	4589383	-1	hypothetical protein
4561321	4597109	35788	L4174_04300	4589380	4589616	-1	hypothetical protein
4561321	4597109	35788	L4174_04301	4589613	4589840	-1	hypothetical protein
4561321	4597109	35788	L4174_04302	4589837	4590286	-1	hypothetical protein
4561321	4597109	35788	L4174_04303	4590283	4590789	-1	hypothetical protein
4561321	4597109	35788	L4174_04304	4590852	4591253	-1	hypothetical protein
4561321	4597109	35788	L4174_04305	4591264	4591788	-1	hypothetical protein
4561321	4597109	35788	L4174_04306	4591918	4592130	-1	hypothetical protein
4561321	4597109	35788	L4174_04307	4592312	4592980	1	hypothetical protein
4561321	4597109	35788	L4174_04308	4593019	4593486	1	hypothetical protein
4561321	4597109	35788	L4174_04309	4593468	4593656	1	hypothetical protein
4561321	4597109	35788	L4174_04310	4593653	4594567	1	hypothetical protein
4561321	4597109	35788	L4174_04311	4594923	4595723	-1	hypothetical protein
4561321	4597109	35788	L4174_04312	4596087	4597109	1	Tyrosine recombinase XerC
4623293	4633504	10211	L4174_04338	4623293	4623661	1	hypothetical protein
4623293	4633504	10211	L4174_04339	4623906	4624403	1	hypothetical protein
4623293	4633504	10211	L4174_04340	4624795	4625616	-1	hypothetical protein
4623293	4633504	10211	L4174_04341	4625698	4626507	-1	hypothetical protein
4623293	4633504	10211	L4174_04342	4626618	4627472	-1	hypothetical protein
4623293	4633504	10211	L4174_04343	4627475	4633504	-1	hypothetical protein
4822763	4835102	12339	L4174_04524	4822763	4823119	1	hypothetical protein

4822763	4835102	12339	L4174_04525	4823437	4823679	-1	hypothetical protein
4822763	4835102	12339	L4174_04526	4823783	4824133	-1	hypothetical protein
4822763	4835102	12339	L4174_04527	4824403	4824693	-1	hypothetical protein
4822763	4835102	12339	L4174_04528	4825652	4826269	-1	hypothetical protein
4822763	4835102	12339	L4174_04529	4827130	4827486	-1	hypothetical protein
4822763	4835102	12339	L4174_04530	4827650	4828204	-1	hypothetical protein
4822763	4835102	12339	L4174_04531	4828407	4828766	-1	hypothetical protein
4822763	4835102	12339	L4174_04532	4829125	4829805	1	Endonuclease V
4822763	4835102	12339	L4174_04533	4829802	4830677	-1	hypothetical protein
4822763	4835102	12339	L4174_04534	4832619	4833182	-1	hypothetical protein
4822763	4835102	12339	L4174_04535	4833435	4834067	-1	hypothetical protein
4822763	4835102	12339	L4174_04536	4834464	4835102	-1	hypothetical protein
4822763	4835102	12339	L4174_04537	4835095	4843794	-1	hypothetical protein
5093778	5105547	11769	L4174_04778	5092876	5093799	-1	Recombination-associated protein RdgC
5093778	5105547	11769	L4174_04779	5093778	5094098	-1	hypothetical protein
5093778	5105547	11769	L4174_04780	5094112	5094363	-1	hypothetical protein
5093778	5105547	11769	L4174_04781	5094468	5094731	-1	hypothetical protein
5093778	5105547	11769	L4174_04782	5094722	5094910	-1	hypothetical protein
5093778	5105547	11769	L4174_04783	5094922	5095179	-1	hypothetical protein
5093778	5105547	11769	L4174_04784	5095410	5095535	-1	hypothetical protein
5093778	5105547	11769	L4174_04785	5095603	5096307	-1	Oligoribonuclease
5093778	5105547	11769	L4174_04786	5096441	5096728	-1	Integration host factor subunit beta
5093778	5105547	11769	L4174_04787	5096718	5097014	-1	Integration host factor subunit alpha
5093778	5105547	11769	L4174_04788	5097157	5097771	-1	hypothetical protein
5093778	5105547	11769	L4174_04789	5097768	5098550	-1	hypothetical protein
5093778	5105547	11769	L4174_04790	5098553	5099944	-1	Chromosome partition protein Smc
5093778	5105547	11769	L4174_04791	5100019	5100276	-1	hypothetical protein
5093778	5105547	11769	L4174_04792	5100278	5100703	-1	hypothetical protein
5093778	5105547	11769	L4174_04793	5100862	5101164	1	hypothetical protein
5093778	5105547	11769	L4174_04794	5101235	5101738	-1	hypothetical protein
5093778	5105547	11769	L4174_04795	5101731	5102630	-1	hypothetical protein
5093778	5105547	11769	L4174_04796	5102906	5103877	-1	hypothetical protein
5093778	5105547	11769	L4174_04797	5103880	5105547	-1	hypothetical protein
5113360	5135615	22255	L4174_04801	5113360	5113665	-1	hypothetical protein
5113360	5135615	22255	L4174_04802	5113948	5114700	1	hypothetical protein
5113360	5135615	22255	L4174_04803	5114757	5114954	1	hypothetical protein
5113360	5135615	22255	L4174_04804	5115198	5116112	-1	Replication protein RepA
5113360	5135615	22255	L4174_04805	5116532	5117767	1	hypothetical protein
5113360	5135615	22255	L4174_04806	5117812	5118150	-1	hypothetical protein
5113360	5135615	22255	L4174_04807	5118199	5120664	-1	hypothetical protein
5113360	5135615	22255	L4174_04808	5120661	5121281	-1	hypothetical protein
5113360	5135615	22255	L4174_04809	5121274	5121696	-1	hypothetical protein
5113360	5135615	22255	L4174_04810	5121693	5121995	-1	hypothetical protein
5113360	5135615	22255	L4174_04811	5122095	5126708	-1	hypothetical protein
5113360	5135615	22255	L4174_04812	5126717	5127142	-1	hypothetical protein
5113360	5135615	22255	L4174_04813	5127155	5127982	-1	hypothetical protein
5113360	5135615	22255	L4174_04814	5127992	5129371	-1	hypothetical protein
5113360	5135615	22255	L4174_04815	5129371	5129709	-1	hypothetical protein
5113360	5135615	22255	L4174_04816	5129715	5132366	-1	hypothetical protein
5113360	5135615	22255	L4174_04817	5132513	5133307	-1	hypothetical protein
5113360	5135615	22255	L4174_04818	5133317	5133919	-1	hypothetical protein
5113360	5135615	22255	L4174_04819	5133926	5134480	-1	hypothetical protein

5113360	5135615	22255	L4174_04820	5134490	5134987	-1	hypothetical protein
5113360	5135615	22255	L4174_04821	5135001	5135615	-1	hypothetical protein
5140212	5151187	10975	L4174_04826	5140212	5140979	-1	hypothetical protein
5140212	5151187	10975	L4174_04827	5141061	5141402	-1	hypothetical protein
5140212	5151187	10975	L4174_04828	5141514	5142077	-1	hypothetical protein
5140212	5151187	10975	L4174_04829	5142029	5142313	-1	hypothetical protein
5140212	5151187	10975	L4174_04830	5142322	5143089	-1	hypothetical protein
5140212	5151187	10975	L4174_04831	5143073	5144071	-1	hypothetical protein
5140212	5151187	10975	L4174_04832	5144068	5144847	-1	hypothetical protein
5140212	5151187	10975	L4174_04833	5144847	5145353	-1	hypothetical protein
5140212	5151187	10975	L4174_04834	5145350	5146519	-1	hypothetical protein
5140212	5151187	10975	L4174_04835	5146512	5146835	-1	hypothetical protein
5140212	5151187	10975	L4174_04836	5146838	5147458	-1	hypothetical protein
5140212	5151187	10975	L4174_04837	5147675	5148352	-1	hypothetical protein
5140212	5151187	10975	L4174_04838	5148477	5148872	-1	hypothetical protein
5140212	5151187	10975	L4174_04839	5148893	5149063	-1	hypothetical protein
5140212	5151187	10975	L4174_04840	5149060	5149512	-1	hypothetical protein
5140212	5151187	10975	L4174_04841	5149509	5150024	-1	hypothetical protein
5140212	5151187	10975	L4174_04842	5150569	5150814	1	hypothetical protein
5140212	5151187	10975	L4174_04843	5150804	5151187	1	hypothetical protein
5140212	5151187	10975	L4174_04844	5151184	5152440	-1	Protein UmuC
5153507	5164127	10620	L4174_04847	5153507	5153854	1	hypothetical protein
5153507	5164127	10620	L4174_04848	5154160	5155383	1	hypothetical protein
5153507	5164127	10620	L4174_04849	5155395	5156195	1	hypothetical protein
5153507	5164127	10620	L4174_04850	5156303	5156617	1	hypothetical protein
5153507	5164127	10620	L4174_04851	5156675	5157514	-1	Modification methylase HpaII
5153507	5164127	10620	L4174_04852	5157565	5158125	-1	hypothetical protein
5153507	5164127	10620	L4174_04853	5158125	5158409	-1	hypothetical protein
5153507	5164127	10620	L4174_04854	5158406	5159437	-1	hypothetical protein
5153507	5164127	10620	L4174_04855	5159620	5159868	-1	hypothetical protein
5153507	5164127	10620	L4174_04856	5159957	5160631	-1	hypothetical protein
5153507	5164127	10620	L4174_04857	5160652	5160903	-1	hypothetical protein
5153507	5164127	10620	L4174_04858	5160914	5161393	-1	hypothetical protein
5153507	5164127	10620	L4174_04859	5161380	5161934	-1	hypothetical protein
5153507	5164127	10620	L4174_04860	5162308	5162565	-1	hypothetical protein
5153507	5164127	10620	L4174_04861	5162576	5163208	-1	Tyrosine recombinase XerC
5153507	5164127	10620	L4174_04862	5163272	5163796	-1	hypothetical protein
5153507	5164127	10620	L4174_04863	5163876	5164127	-1	hypothetical protein

Table S4. Prophage elements detected in the CCB-ST2H9 genome by PHASTER [35].

Location	Start	End	Length (Kb)	Completeness (score)	Hit proteins
Contig 1	2,760,651	2,782,440	21.7	Incomplete (30)	9
Contig 2	512,459	518,503	6	Intact (107)	9
Contig 2	972,058	1,022,714	50.6	Questionable (90)	60
Contig 3	27,911	62,561	34.6	Incomplete (20)	36
Contig 3	66,373	76,226	9.8	Incomplete (20)	13

Table S5. digital DNA-DNA hybridization (dDDH) values between *Photobacterium* genomes.

	P. alginatilyticum	*P. andalusiense*	*P. angustum*	*P. aphoticum*	*P. aquae*	*P. aquimaris*	*P. arenosum*	*P. carnosum*	*P. chitinilyticum*	*P. damselae*	*P. frigidiphilum*	*P. gaetbulicola*	*P. galatheae*	*P. ganghwense*	*P. halotolerans*	*P. iliopiscarium*	*P. indicum*	*P. jeanii*	*P. kishitanii*	*P. leiognathi*	*P. lipolyticum*	*P. lucens*	*P. lutimaris*	*P. malacitanum*	*P. mandapamensis*	*P. marinum*	*P. phosphoreum*	*P. piscicola*	*P. profundum*	*P. proteolyticum*	*P. rosenbergii*	*P. salinisoli*	*P. sanctipauli*	*P. sanguinicancri*	*P. swingsii*	*P. toruni*	CCB-ST2H9
P. alginatilyticum	100	20	19.9	21.3	20.7	19.6	20.4	19.4	25.3	20.8	19.6	21.7	20.4	21.4	20.3	19.5	19.5	20.7	19.5	20.4	21.2	20.7	21.2	20.3	20.2	21.5	19.5	19.9	19.7	52.3	21.2	20.7	21.3	21.1	20.3	19.9	20.3
P. andalusiense	20	100	21.2	20.6	20.5	62.9	19.7	27.4	20.2	21.6	20.8	21.3	20.9	21.8	19.3	27.1	20.5	20.4	29.4	21	20.4	21	20.4	50.3	20.9	20	29.2	27.2	21.7	20	20.5	20.4	20.4	21	21	28.2	20.8
P. angustum	19.9	21.2	100	21.2	20	21.1	19.7	20.8	20.5	21.7	20.1	20.8	20.1	21.8	19.6	20.9	20.1	21	21	25.3	20.2	24.8	20.8	21.4	24.9	20	21	21.3	21.4	19.8	21	19.5	20.8	20.9	21	21	20.3
P. aphoticum	21.3	20.6	21.2	100	21.8	20.2	20.8	20.2	20.9	22.4	20	21.6	21	21.8	20.4	20.4	20	21.6	20.5	21.7	21	21.3	21.2	20.4	21.2	21.5	20.2	20.6	20.6	21.5	21.5	20.8	21.4	21	21.2	20.6	20.7
P. aquae	20.7	20.5	20	21.8	100	20	20	20	19.9	22.4	19.9	21	20.2	21.6	19.7	19.5	19.8	20.7	20.1	21.1	20.6	20.6	20.6	20.5	20.8	20.7	19.6	20.5	20.8	20.6	20.9	19.9	20.9	20.2	20.2	20.5	20.1
P. aquimaris	19.6	62.9	21.1	20.2	20	100	19.4	28.1	19.7	21.1	20.5	20.3	19.6	20.4	19.3	26.8	20.3	20.3	29.6	20.8	20	20.9	20.3	49.6	20.8	19.5	28.9	26.8	21	19.6	20.2	19.3	19.8	20.5	20.4	28.1	19.6
P. arenosum	20.4	19.7	19.7	20.8	20	19.4	100	20.4	20	21.8	19.3	20.9	22.2	22.1	52.4	20.2	19.2	19.9	19.6	19.8	20	19.8	20.5	19.9	19.7	20	20	20.7	20.3	20.4	20.3	42.5	19.6	19.3	19.5	20.2	22.1
P. carnosum	19.4	27.4	20.8	20.2	20	28.1	20.4	100	20	21.3	20.4	20.8	20.6	21.5	20	44.8	20.5	20.7	28.7	20.6	19.8	20.5	20.4	26.9	20.7	19.3	30.8	34.8	21.5	19.5	20.2	20.4	20.2	20.5	20.6	33.1	20.2
P. chitinilyticum	25.3	20.2	20.5	20.9	19.9	19.7	20	20	100	21.4	20.2	20.8	19.9	21	20	20	20.5	20.7	20.1	20.3	21.4	20.5	20.7	20.2	20.1	21	19.8	20.4	20.5	25.1	20.6	20.2	20.6	20.6	21	20.6	19.9
P. damselae	20.8	21.6	21.7	22.4	22.4	21.1	21.8	21.3	21.4	100	20.9	23.3	23.1	24.4	20	21.2	21	22.5	21.1	22.2	21	22.3	22.3	22.2	21.5	21.8	20.9	21.5	22.1	20.7	22.8	21.8	22.4	21.9	21.9	21.2	23.3
P. frigidiphilum	19.6	20.8	20.1	20	19.9	20.5	19.3	20.4	20.2	20.9	100	19.7	19.6	20.5	19	20.2	56.3	20.3	20.7	20.1	22.2	20.3	19.8	20.7	20.3	19.6	20.1	20.8	63.8	19.6	19.7	19.6	19.6	21.2	20.8	20.5	19.5
P. gaetbulicola	21.7	21.3	20.8	21.6	21	20.3	20.9	20.8	20.8	23.3	19.7	100	21.2	22.3	20.6	20.7	19.5	21.2	20.6	21.3	20.7	21.6	24.7	21.2	20.8	21.5	20.6	21	20.4	21.6	26.3	21.2	22	20.9	21	20.6	21.3
P. galatheae	20.4	20.9	20.1	21	20.2	19.6	22.2	20.6	19.9	23.1	19.6	21.2	100	23.3	22.1	19.9	19.7	20.5	20.2	20.1	20.3	20.1	20.4	20.7	19.9	20.5	19.8	20.5	20.8	20.6	20.7	22.2	20.1	19.9	20.2	20.7	32.5
P. ganghwense	21.4	21.8	21.8	21.8	21.6	20.4	22.1	21.5	21	24.4	20.5	22.3	23.3	100	21.1	20.8	20	21.4	21.1	22.1	21.4	22.2	21.5	21.6	21.1	22.1	20.8	22.1	21.4	21.6	21.6	22.3	21.5	21.1	21.3	21.7	23.1
P. halotolerans	20.3	19.3	19.6	20.4	19.7	19.3	52.4	20	20	20	19	20.6	22.1	21.1	100	20	18.9	19.8	19	19.6	19.9	19.4	20.1	19.3	19.5	20	19.3	20.1	19.1	20.4	20.3	42.5	19.8	19.1	19.4	19.5	22.1
P. iliopiscarium	19.5	27.1	20.9	20.4	19.5	26.8	20.2	44.8	20	21.2	20.2	20.7	19.9	20.8	20	100	20.3	20.4	28.1	20.8	19.8	20.7	20.2	26.4	20.5	19.8	30.2	38.6	21	19.3	19.9	20.2	20	20.8	20.9	31.5	19.7
P. indicum	19.5	20.5	20.1	20	19.8	20.3	19.2	20.5	20.5	21	56.3	19.5	19.7	20	18.9	20.3	100	20.2	20.6	20.4	21.7	20.4	19.8	20.7	20.5	19.6	20.4	20.8	54.5	19.5	19.5	19.3	19.8	21.2	20.9	20.8	19.4
P. jeanii	20.7	20.4	21	21.6	20.7	20.3	19.9	20.7	20.7	22.5	20.3	21.2	20.5	21.4	19.8	20.4	20.2	100	20.6	21.1	20.7	21	21.1	20.7	20.9	20.9	20.3	20.7	20.6	20.7	21.4	20.1	21.3	21.8	22.1	20.4	20.2
P. kishitanii	19.5	29.4	21	20.5	20.1	29.6	19.6	28.7	20.1	21.1	20.7	20.6	20.2	21.1	19	28.1	20.6	20.6	100	20.9	20	20.7	20.4	28.9	20.7	19.6	30.6	27.8	21.4	19.6	20.3	19.6	20.4	20.6	20.5	28.6	20
P. leiognathi	20.4	21	25.3	21.7	21.1	20.8	19.8	20.6	20.3	22.2	20.1	21.3	20.1	22.1	19.6	20.8	20.4	21.1	20.9	100	20.1	48.1	20.9	21.3	73.1	20.5	20.9	21	21.1	20.1	21	19.7	21	21	21	21.3	20.1
P. lipolyticum	21.2	20.4	20.2	21	20.6	20	20	19.8	21.4	21	22.2	20.7	20.3	21.4	19.9	19.8	21.7	20.7	20	20.1	100	20.3	21	20.3	20.1	20.4	19.7	20.3	22.4	21	20.3	20.1	20.6	20.9	20.8	20.2	20.3

P. lucens	20.7	21	24.8	21.3	20.6	20.9	19.8	20.5	20.5	22.3	20.3	21.6	20.1	22.2	19.4	20.7	20.4	21	20.7	48.1	20.3	100	20.8	21.2	46.7	20.4	20.9	20.9	20.9	20.5	21.1	19.7	21.6	20.9	21.1	21	20.2
P. lutimaris	21.2	20.4	20.8	21.2	20.6	20.3	20.5	20.4	20.7	22.3	19.8	24.7	20.4	21.5	20.1	20.2	19.8	21.1	20.4	20.9	21	20.8	100	20.7	20.7	21.1	20.5	20.8	20.4	21.1	24.1	20.4	21.5	20.8	21	20.2	20.3
P. malacitanum	20.3	50.3	21.4	20.4	20.5	49.6	19.9	26.9	20.2	22.2	20.7	21.2	20.7	21.6	19.3	26.4	20.7	20.7	28.9	21.3	20.3	21.2	20.7	100	21	20	28.3	26.4	21.3	20.1	20.5	20.3	20.2	21.2	20.9	27.5	20.6
P. mandapamensis	20.2	20.9	24.9	21.2	20.8	20.8	19.7	20.7	20.1	21.5	20.3	20.8	19.9	21.1	19.5	20.5	20.5	20.9	20.7	73.1	20.1	46.7	20.7	21	100	20.6	20.6	20.8	20.6	20.2	20.8	19.6	21	20.5	21.1	20.9	19.7
P. marinum	21.5	20	20	21.5	20.7	19.5	20	19.3	21	21.8	19.6	21.5	20.5	22.1	20	19.8	19.6	20.9	19.6	20.5	20.4	20.4	21.1	20	20.6	100	20	20.2	20.1	21.4	21.4	20.1	20.9	20	20.3	19.8	20.4
P. phosphoreum	19.5	29.2	21	20.2	19.6	28.9	20	30.8	19.8	20.9	20.1	20.6	19.8	20.8	19.3	30.2	20.4	20.3	30.6	20.9	19.7	20.9	20.5	28.3	20.6	20	100	29	20.9	19.6	20.2	20.3	20	20.5	20.4	29.2	19.7
P. piscicola	19.9	27.2	21.3	20.6	20.5	26.8	20.7	34.8	20.4	21.5	20.8	21	20.5	22.1	20.1	38.6	20.8	20.7	27.8	21	20.3	20.9	20.8	26.4	20.8	20.2	29	100	21.5	19.7	20.5	21.1	21	20.9	20.9	32.6	20.6
P. profundum	19.7	21.7	21.4	20.6	20.8	21	20.3	21.5	20.5	22.1	63.8	20.4	20.8	21.4	19.1	21	54.5	20.6	21.4	21.1	22.4	20.9	20.4	21.3	20.6	20.1	20.9	21.5	100	19.6	20.5	20.5	20.2	21.9	21.7	21.4	20.9
P. proteolyticum	52.3	20	19.8	21.5	20.6	19.6	20.4	19.5	25.1	20.7	19.6	21.6	20.6	21.6	20.4	19.3	19.5	20.7	19.6	20.1	21	20.5	21.1	20.1	20.2	21.4	19.6	19.7	19.6	100	21.2	20.4	21.3	20.6	20.4	19.8	20.3
P. rosenbergii	21.2	20.5	21	21.5	20.9	20.2	20.3	20.2	20.6	22.8	19.7	26.3	20.7	21.6	20.3	19.9	19.5	21.4	20.3	21	20.3	21.1	24.1	20.5	20.8	21.4	20.2	20.5	20.5	21.2	100	20.4	21.5	20.7	20.6	20.3	20.7
P. salinisoli	20.7	20.4	19.5	20.8	19.9	19.3	42.5	20.4	20.2	21.8	19.6	21.2	22.2	22.3	42.5	20.2	19.3	20.1	19.6	19.7	20.1	19.7	20.4	20.3	19.6	20.1	20.3	21.1	20.5	20.4	20.4	100	19.9	19.3	19.5	20.2	21.8
P. sanctipauli	21.3	20.4	20.8	21.4	20.9	19.8	19.6	20.2	20.6	22.4	19.6	22	20.1	21.5	19.8	20	19.8	21.3	20.4	21	20.6	21.6	21.5	20.2	21	20.9	20	21	20.2	21.3	21.5	19.9	100	20.5	20.9	20.2	20.4
P. sanguinicancri	21.1	21	20.9	21	20.2	20.5	19.3	20.5	20.6	21.9	21.2	20.9	19.9	21.1	19.1	20.8	21.2	21.8	20.6	21	20.9	20.9	20.8	21.2	20.5	20	20.5	20.9	21.9	20.6	20.7	19.3	20.5	100	28.3	21	19.6
P. swingsii	20.3	21	21	21.2	20.2	20.4	19.5	20.6	21	21.9	20.8	21	20.2	21.3	19.4	20.9	20.9	22.1	20.5	21	20.8	21.1	21	20.9	21.1	20.3	20.4	20.9	21.7	20.4	20.6	19.5	20.9	28.3	100	20.8	19.7
P. toruni	19.9	28.2	21	20.6	20.5	28.1	20.2	33.1	20.6	21.2	20.5	20.6	20.7	21.7	19.5	31.5	20.8	20.4	28.6	21.3	20.2	21	20.2	27.5	20.9	19.8	29.2	32.6	21.4	19.8	20.3	20.2	20.2	21	20.8	100	20.3
CCB-ST2H9	20.6	22.3	21.4	21.1	21	20.4	22.3	21.8	20.5	24.4	20.6	21.9	32.7	23.6	22.2	21.0	20.2	20.9	21.2	21.3	21.1	21.3	20.9	22.1	20.2	21	20.7	22	21.8	20.5	21.4	22	21.2	20.5	20.6	21.6	100

Table S6. Biosynthetic gene clusters detected in *Photobacterium* genomes.

	arylpolyene	arylpolyene,ectoine	betalactone	butyrolactone	cyanobactin	ectoine	hserlactone	lanthipeptide-class-i	NRPS	NRPS-like	NRPS, NRPS-like	NRPS,transAT-PKS	NRPK,TIPKS	NRPS,NRPS-like,T1PKS	phenazine	PKS	PUFA,hglE-KS	RiPP	RiPP-like	RiPP-like,arylpolyene,resorcinol	RRE-containing	siderophore	terpene	thioamitides	thiopeptide	T1PKS	T3PKS
P. alginatilyticum	1		1				1		3	1		1					1		1					1			
P. andalusiense	1		1																1								
P. angustum		1	1																1			1	1				
P. aphoticum		1	1			1				1									1			1			1		
P. aquae	1		1			1				1									1						1		
P. aquimaris	1		1															1									
P. arenosum			1			1	1	1	4		1				1	1			1			1					
P. carnosum			1																1								
P. chitinilyticum	2		1				1			1		1	1				1		1								
P. damselae			1																						1		
P. frigidiphilum	1		1	1		1			1	1							1		1						1		
P. gaetbulicola			1							1		1		1					2						1		
P. galatheae			1			1			5	3				2					1			1		1			
P. ganghwense		1	1			1			1	1									1						1	1	
P. halotolerans			1			1			3	1					1				1			1					1
P. iliopiscarium			1																1								
P. indicum	1		1			1			1	1			1				1		1	1					1		
P. jeanii	1		1																1			1			1		

P. kishtanii	1		1																1								
P. leiognathi		1	1																1								
P. lipolyticum	1					1			1								1	2									
P. lucens		1	1																1			1					
P. lutimaris	1		1							1		1	1						1						1		
P. malacitanum	1		1																1								
P. mandapamensis		1	1															1									
P. marinum			1							1			1						1						1		
P. phosphoreum	1		1																1			1					
P. piscicola	1		1																1								
P. profundum	1		1						2					1			1	1							1		
P. proteolyticum	1		1				1		3	1		1					1		1					1			
P. rosenbergii			1							1									2						1		
P. salinisoli			1			1	1		2							1			1								
P. sanctipauli			1															3				1					
P. sanguinicancri	1								1								1		1			1					
P. swingsii	1		1						1								1		1		1	1					
P. toruni			1																1								
CCB-ST2H9			1	1	1	1			2	1	1								1			1					

References

1. Wang, X.; Wang, Y.; Yang, X.; Sun, H.; Li, B.; Zhang, X.H. *Photobacterium alginatilyticum* sp. nov., a marine bacterium isolated from bottom seawater. *Int. J. Syst. Evol. Microbiol.* **2017**, *67*, 1912-1917.
2. Labella, A.M.; Castro, M.D.; Manchado, M.; Lucena, T.; Arahal, D.R.; Borrego, J.J. *Photobacterium malacitanum* sp. nov., and *Photobacterium andalusiense* sp. nov., two new bacteria isolated from diseased farmed fish in Southern Spain. *Syst. Appl. Microbiol.* **2018**, *41*, 444-451.
3. Reichelt, J.L.; Baumann, P.; Baumann, L. Study of genetic relationships among marine species of the genera *Beneckea* and *Photobacterium* by means of in vitro DNA/DNA hybridization. *Arch. Microbiol.* **1976**, *110*, 101-120.
4. Lucena, T.; Ruvira, M.A.; Pascual, J.; Garay, E.; Macián, M.C.; Arahal, D.R.; Pujalte, M.J. *Photobacterium aphoticum* sp. nov., isolated from coastal water. *Int. J. Syst. Evol. Microbiol.* **2011**, *61*, 1579-1584.
5. Liu, Y.; Liu, L.Z.; Song, L.; Zhou, Y.G.; Qi, F.J.; Liu, Z.P. *Photobacterium aquae* sp. nov., isolated from a recirculating mariculture system. *Int. J. Syst. Evol. Microbiol.* **2014**, *64*, 475-480.
6. Yoshizawa, S.; Wada, M.; Kita-Tsukamoto, K.; Yokota, A.; Kogure, K. *Photobacterium aquimaris* sp. nov., a luminous marine bacterium isolated from seawater. *Int. J. Syst. Evol. Microbiol.* **2009**, *59*, 1438-1442.
7. Weerawongwiwat, V.; Yoon, S.; Kim, J.-H.; Yoon, J.-H.; Lee, J.S.; Sukhoom, A.; Kim, W. *Photobacterium arenosum* sp. nov., isolated from marine sediment sand. **2021**, *71*, 005034.

8. Hilgarth, M.; Fuertes, S.; Ehrmann, M.; Vogel, R.F. *Photobacterium carnosum* sp. nov., isolated from spoiled modified atmosphere packaged poultry meat. *Syst. Appl. Microbiol.* **2018**, *41*, 44-50.

9. Wang, X.; Li, Y.; Xue, C.X.; Li, B.; Zhou, S.; Liu, L.; Zhang, X.H. *Photobacterium chitinilyticum* sp. nov., a marine bacterium isolated from seawater at the bottom of the East China Sea. *Int. J. Syst. Evol. Microbiol.* **2019**, *69*, 1477-1483.

10. Lee, K.; Kim, H.K.; Sohn, H.; Cho, Y.; Choi, Y.M.; Jeong, D.G.; Kim, J.H. Genomic insights into *Photobacterium damselae* subsp. damselae strain KC-Na-1, isolated from the finless porpoise (*Neophocaena asiaeorientalis*). *Mar. Genomics* **2018**, *37*, 26-30.

11. Seo, H.J.; Bae, S.S.; Lee, J.H.; Kim, S.J. *Photobacterium frigidiphilum* sp. nov., a psychrophilic, lipolytic bacterium isolated from deep-sea sediments of Edison Seamount. *Int. J. Syst. Evol. Microbiol.* **2005**, *55*, 1661-1666.

12. Kim, Y.O.; Kim, K.K.; Park, S.; Kang, S.J.; Lee, J.H.; Lee, S.J.; Oh, T.K.; Yoon, J.H. *Photobacterium gaetbulicola* sp. nov., a lipolytic bacterium isolated from a tidal flat sediment. *Int. J. Syst. Evol. Microbiol.* **2010**, *60*, 2587-2591.

13. Machado, H.; Giubergia, S.; Mateiu, R.V.; Gram, L. *Photobacterium galatheae* sp. nov., a bioactive bacterium isolated from a mussel in the Solomon Sea. *Int. J. Syst. Evol. Microbiol.* **2015**, *65*, 4503-4507.

14. Lascu, I.; Mereuță, I.; Chiciudean, I.; Hansen, H.; Avramescu, S.M.; Tănase, A.M.; Stoica, I. Complete genome sequence of *Photobacterium ganghwense* C2.2: A new polyhydroxyalkanoate production candidate. *MicrobiologyOpen* **2021**, *10*, e1182.

15. Rivas, R.; García-Fraile, P.; Mateos, P.F.; Martínez-Molina, E.; Velázquez, E. *Photobacterium halotolerans* sp. nov., isolated from Lake Martel in Spain. *Int. J. Syst. Evol. Microbiol.* **2006**, *56*, 1067-1071.

16. Urakawa, H.; Kita-Tsukamoto, K.; Ohwada, K. Reassessment of the taxonomic position of *Vibrio iliopiscarius* (Onarheim et al. 1994) and proposal for *Photobacterium iliopiscarium* comb. nov. *Int. J. Syst. Bacteriol.* **1999**, *49,* 257-260.

17. Xie, C.H.; Yokota, A. Transfer of *Hyphomicrobium indicum* to the genus *Photobacterium* as *Photobacterium indicum* comb. nov. *Int. J. Syst. Evol. Microbiol.* **2004**, *54*, 2113-2116.

18. Chimetto, L.A.; Cleenwerck, I.; Thompson, C.C.; Brocchi, M.; Willems, A.; De Vos, P.; Thompson, F.L. *Photobacterium jeanii* sp. nov., isolated from corals and zoanthids. *Int. J. Syst. Evol. Microbiol.* **2010**, *60*, 2843-2848.

19. Ast, J.C.; Cleenwerck, I.; Engelbeen, K.; Urbanczyk, H.; Thompson, F.L.; De Vos, P.; Dunlap, P.V. *Photobacterium kishitanii* sp. nov., a luminous marine bacterium symbiotic with deep-sea fishes. *Int. J. Syst. Evol. Microbiol.* **2007**, *57*, 2073-2078.

20. Urbanczyk, H.; Urbanczyk, Y.; Hayashi, T.; Ogura, Y. Diversification of two lineages of symbiotic *Photobacterium*. *PLoS One* **2013**, *8*, e82917.

21. Yoon, J.H.; Lee, J.K.; Kim, Y.O.; Oh, T.K. *Photobacterium lipolyticum* sp. nov., a bacterium with lipolytic activity isolated from the Yellow Sea in Korea. *Int. J. Syst. Evol. Microbiol.* **2005**, *55*, 335-339.

22. Enciso-Ibarra, J.; González-Castillo, A.; Soto-Rodriguez, S.A.; Enciso-Ibarra, K.; Bolán-Mejia, C.; Gomez-Gil, B. *Photobacterium lucens* sp. nov., isolated from a cultured shrimp *Penaeus vannamei*. *Curr Microbiol* **2020**, *77*, 1111-1116.

23. Jung, S.Y.; Jung, Y.T.; Oh, T.K.; Yoon, J.H. *Photobacterium lutimaris* sp. nov., isolated from a tidal flat sediment in Korea. *Int. J. Syst. Evol. Microbiol.* **2007**, *57*, 332-336.

24. Srinivas, T.N.; Vijaya Bhaskar, Y.; Bhumika, V.; Anil Kumar, P. *Photobacterium marinum* sp. nov., a marine bacterium isolated from a sediment sample from Palk Bay, India. *Syst. Appl. Microbiol.* **2013**, *36*, 160-165.
25. Figge, M.J.; Cleenwerck, I.; van Uijen, A.; De Vos, P.; Huys, G.; Robertson, L. *Photobacterium piscicola* sp. nov., isolated from marine fish and spoiled packed cod. *Syst. Appl. Microbiol.* **2014**, *37*, 329-335.
26. DeLong, E.F.; Franks, D.G.; Yayanos, A.A. Evolutionary relationships of cultivated psychrophilic and barophilic deep-sea bacteria. *Appl. Environ. Microbiol.* **1997**, *63*, 2105-2108.
27. Li, Y.; Zhou, M.; Wang, F.; Wang, E.T.; Du, Z.; Wu, C.; Zhang, Z.; Liu, W.; Xie, Z. *Photobacterium proteolyticum* sp. nov., a protease-producing bacterium isolated from ocean sediments of Laizhou Bay. *Int. J. Syst. Evol. Microbiol.* **2017**, *67*, 1835-1840.
28. Thompson, F.L.; Thompson, C.C.; Naser, S.; Hoste, B.; Vandemeulebroecke, K.; Munn, C.; Bourne, D.; Swings, J. *Photobacterium rosenbergii* sp. nov. and *Enterovibrio coralii* sp. nov., vibrios associated with coral bleaching. *Int. J. Syst. Evol. Microbiol.* **2005**, *55*, 913-917.
29. Li, M.; Kong, D.; Wang, Y.; Ma, Q.; Han, X.; Zhou, Y.; Jiang, X.; Zhang, Y.; Ruan, Z.; Zhang, Q. *Photobacterium salinisoli* sp. nov., isolated from a sulfonylurea herbicide-degrading consortium enriched with saline soil. *Int. J. Syst. Evol. Microbiol.* **2019**, *69*, 3910-3916.
30. Moreira, A.P.B.; Duytschaever, G.; Chimetto Tonon, L.A.; Fróes, A.M.; de Oliveira, L.S.; Amado-Filho, G.M.; Francini-Filho, R.B.; De Vos, P.; Swings, J.; Thompson, C.C.; et al. *Photobacterium sanctipauli* sp. nov. isolated from bleached *Madracis decactis* (Scleractinia) in the St Peter & St Paul Archipelago, Mid-Atlantic Ridge, Brazil. *PeerJ* **2014**, *2*, e427.

31. Gomez-Gil, B.; Roque, A.; Rotllant, G.; Romalde, J.L.; Doce, A.; Eggermont, M.; Defoirdt, T. *Photobacterium sanguinicancri* sp. nov. isolated from marine animals. *Antonie Van Leeuwenhoek* **2016**, *109*, 817-825.

32. Gomez-Gil, B.; Roque, A.; Rotllant, G.; Peinado, L.; Romalde, J.L.; Doce, A.; Cabanillas-Beltrán, H.; Chimetto, L.A.; Thompson, F.L. *Photobacterium swingsii* sp. nov., isolated from marine organisms. *Int. J. Syst. Evol. Microbiol.* **2011**, *61*, 315-319.

33. Labella, A.M.; Arahal, D.R.; Lucena, T.; Manchado, M.; Castro, D.; Borrego, J.J. *Photobacterium toruni* sp. nov., a bacterium isolated from diseased farmed fish. *Int. J. Syst. Evol. Microbiol.* **2017**, *67*, 4518-4525.

34. Bertelli, C.; Laird, M.R.; Williams, K.P.; Simon Fraser University Research Computing Group; Lau, B.Y.; Hoad, G.; Winsor, G.L.; Brinkman, F.S.L. IslandViewer 4: expanded prediction of genomic islands for larger-scale datasets. *Nucleic Acids Res.* **2017**, *45*, W30-W35.

35. Arndt, D.; Grant, J.R.; Marcu, A.; Sajed, T.; Pon, A.; Liang, Y.; Wishart, D.S. PHASTER: a better, faster version of the PHAST phage search tool. *Nucleic Acids Res.* **2016**, *44*, W16-W21.
